# Early Identification of Progressive TB Disease Using Host Biomarkers

**DOI:** 10.1016/j.ebiom.2015.01.009

**Published:** 2015-01-16

**Authors:** Andrea Rachow, Norbert Heinrich, Christof Geldmacher

**Affiliations:** Department of Infectious Diseases and Tropical Medicine, Medical Centre of the University of Munich (LMU), Munich, Germany; German Centre for Infection Research (DZIF), Partner Site Munich, Munich, Germany

Identification and evaluation of diagnostic biomarkers that predict TB disease progression are elaborate and complicated undertakings, since the number of incident cases even in high risk study populations is usually low and the definition of latent M.tb infection through a positive TST or IGRA test result is inaccurate. Using a retrospective case–control study approach embedded in the *Amsterdam Cohort Studies on HIV infection and AIDS* (van den Hoek et al., 1988), Sloot et al. (in press) identify a transcriptional signature within Peripheral Blood Mononuclear Cells, which identifies HIV-positive subjects who are later diagnosed with active TB disease. 55 to 250 days prior to TB diagnosis, IL13 transcription was detected in six out of 14 *TB progressors* and linked to differential expression of 50 additional genes, including type I IFN signature genes, while no subject in the control group expressed IL13. It can be assumed that this signature is part of an early host immune response to mycobacterial replication in vivo. Whether it is a hallmark of deficient immunological control of the pathogen invariably linked to progressive disease or rather reflective of pathogen activity independent of subsequent disease progression remains unclear.

The study by Sloot et al. supports the concept that a diagnostic approach based on host biomarkers cannot only improve the diagnosis of active TB in populations where a conventional sputum-based diagnosis is difficult (e.g. in children, HIV-positives, extra-pulmonary TB), but might further allow identification of subjects with sub-clinically active M.tb replication. Other recent articles also highlighted the potential of host biomarkers based on transcriptional (Anderson et al., 2014, Joosten et al., 2012) or novel *T cell* signatures (Portevin et al., 2014, Harari et al., 2011, Schuetz et al., 2011) to improve diagnosis of active TB and possibly also allow early detection of TB disease progression. It is widely accepted that often a substantial time interval exists between infection with M.tb, and diagnosis of symptomatic disease. Individuals who develop active TB disease after primary infection frequently do so within the first two years (Marais et al., 2004). This specific characteristic of M.tb infection provides a window of opportunity for more accurate identification of individuals currently progressing to active TB using such novel biomarkers and to target them for early therapeutic intervention. Particularly in high risk populations such as HIV-positives, TB-contacts or IFN-gamma release assay converters (or TST skin-test converters) this approach might be more attractive than the current suboptimal algorithms with very limited specificity to trigger isoniazid preventive treatment, which likely result in overtreatment (American Thoracic Society, 2000). The use of novel host biomarkers in such a strategy would not only help to significantly reduce TB disease morbidity by detection of those subjects with the highest risk of active TB development in a “pre-clinical” stadium and motivating them and the treating medical staff to adhere to preventive treatment, but could also help to abrogate the MTB transmission chain and hence contribute to the EndTB Strategy by WHO and to the vision of a world free of TB in 2050 (WHO, 2014).

Future research in the field of *host biomarkers predictive of TB disease* should be aiming at simplifying current diagnostic approaches into affordable products applicable on a large scale. Simultaneously, prospective large scale studies on early detection of progressive TB disease will be necessary (even though complicated to conduct) to further confirm the diagnostic accuracy and feasibility of these novel biomarkers in the field. Especially, the specificity for progressive versus non-progressive/latent M.tb infection needs to be assessed, as well as the biomarkers ability to distinguish between TB and other inflammatory diseases. An assessment of host biomarkers would require validation work in patient populations with different genetic backgrounds, as ethnicity can influence the composition of the host response, making such studies even larger. Significant effort is still required until the application of such novel diagnostic strategies on a large scale could be achieved. Nonetheless, new diagnostic host markers for early identification of progressive TB disease might herald a new era in TB diagnosis and early treatment.

## Conflicts of interest

The authors declared no conflicts of interest.
